# The Landscape and Management of Brain Parenchymal and Leptomeningeal Metastases in EGFR Mutated Non-Small Cell Lung Cancer

**DOI:** 10.3390/cancers17152434

**Published:** 2025-07-23

**Authors:** Jonathan Hyak, Sawsan Rashdan

**Affiliations:** Division of Hematology and Oncology, The University of Texas Southwestern, Dallas, TX 75390, USA; sawsan.rashdan@utsouthwestern.edu

**Keywords:** EGFR, leptomeningeal disease, lung adenocarcinoma, lung cancer, brain metastases, targeted therapy

## Abstract

Central nervous system metastases involving the brain parenchyma and leptomeninges are common in non-small cell lung cancer, especially cases with *EGFR* mutations. Here, we examine treatment options for *EGFR*-mutated non-small cell lung cancer patients with central nervous system metastases, highlighting the efficacy of third-generation *EGFR*-targeted tyrosine kinase inhibitors. Furthermore, we examine the interplay of this modality with chemotherapy or radiation in resistant cases.

## Simple Summary

Spread of lung cancer to the brain and lining of the central nervous system is a devastating consequent of disease and is especially common with *EGFR* mutations. Here, we explore the existing literature on disease patterns and management of central nervous system spread for *EGFR*-mutated non-small-cell lung cancer.

## 1. Introduction

Central nervous system (CNS) metastases are present in some 14% of non-small cell lung cancer (NSCLC) patients at diagnosis, with nearly 40% of patients developing CNS metastases at some point in their disease course [1,2]. The presence of brain metastases (BMs), unsurprisingly, leads to worse median overall survival [1]. Among NSCLC cases with activating mutations in *EGFR*—a tyrosine kinase which activates cell growth and proliferation—multiple studies have shown the incidence of CNS metastasis is enriched, with rates some 3–32% higher than *EGFR* wild-type cases (*EGFR^wt^*) [2,3,4,5,6]. Of the two most common *EGFR* abnormalities, BM rates may be even higher with exon 19 deletions than L858R mutations [5].

Interestingly, the presence of BMs may not affect survival in *EGFR^mut^* patients, and *EGFR^mut^* might represent an independent positive predictor of survival in NSCLC patients with BMs, though these findings certainly have not been universal [5,7,8,9,10,11,12]. One potential explanation is the emergence of tyrosine kinase inhibitors (TKIs) targeted against *EGFR^mut^*, which improve outcomes compared to chemotherapy alone [9]. Discrepancies in survival outcomes in *EGFR^mut^* patients with BMs treated with these targeted therapies may reflect the pharmacokinetic properties of different *EGFR^mut^*-specific TKIs and their ability to cross the blood–brain barrier.

Leptomeningeal disease (LMD), a devastating complication of metastatic NSCLC, also occurs more frequently in *EGFR^mut^* patients, occurring in in some 9.4% of patients compared to 1.7% of *EGFR^wt^* [5,13]. While survival with LMD was historically limited to 1–3 months, targeting *EGFR^mut^* has shown promise in significantly extended survival [13,14,15]. What is less clear is the role of more traditional approaches to LMD, such as radiation and intrathecal chemotherapy, in the age of TKIs.

Herein, we discuss the role of *EGFR*-directed therapy in outcomes for NSCLC patients with BMs. Further, we explore treatment approaches after progression on TKI and what existing studies can tell us about continuing TKI therapy in patients who have non-CNS progression while on TKI therapy.

## 2. Body

### 2.1. Brain Metastases

#### 2.1.1. First- and Second-Generation EGFR TKIs

A meta-analysis by Li, et al. found that the presence of an *EGFR* mutation confers a survival benefit to NSCLC patients with BMs, which may be explained by their finding that use of *EGFR^mut^*-targeted TKIs significantly improved survival in patients with BMs [8]. Though this suggests a mitigating effect of TKI therapy on NSCLC with BMs, this study does not specify which specific TKIs were used and what their individual impact was on survival. Individual studies of the first- and second-generation TKIs erlotinib, gefitinib, and afatinib suggest a more nuanced situation.

In a small, non-comparative retrospective study of gefitinib, the presence of BMs was not a significant predictor of overall survival in multivariate analysis [16]. Optimistically, this suggests first-generation targeted therapy negates the presumed survival detriment imbued by BMs; however, larger studies still show that BMs portend worse outcomes, despite first-generation TKI therapy [10,11]. Furthermore, there was no significant benefit to erlotinib in the subgroups with and without CNS metastases in a phase II study [17].

In multi-modality studies, erlotinib, even when administered concomitantly with whole-brain radiation (WBRT), is outperformed by stereotactic radiosurgery (SRS) alone, and outcomes with gefitinib are improved with the addition of chemotherapy [18,19]. In an attempt to increase the efficacy of erlotinib for BMs, a “pulsatile” regimen of up to 1500 mg of weekly erlotinib was administered in six patients. The best response was partial response in four, stable disease in one, and progressive disease in two [20]. There are other case reports and case studies exploring this “pulsatile” technique, but no prospective trials, making this an experimental treatment method.

Combined analysis of the LUX-Lung 3 and LUX-Lung 6 studies, in which the second-generation *EGFR^mut^* TKI afatinib was compared to chemotherapy and patients with BMs were a pre-specified subgroup, the survival benefit with afatinib in the overall cohort was not demonstrated amongst patients with BMs, and there was no improvement in rates of CNS progression [21]. This lack of CNS efficacy has been demonstrated in real-world data and large retrospective cohorts, as well [10,11,12]. The picture is muddied by a large retrospective cohort comparing CNS efficacy of erlotinib, gefitinib, and afatinib, which demonstrated improved CNS progression-free survival (PFS) with afatinib, compared to erlotinib and gefitinib, in multivariate analysis adjusting for potential confounders, such as age (the afatinib cohort was younger) and *EGFR* exon 19 deletions (higher rates in afatinib cohort) [22]. Results favoring erlotinib over gefitinib and afatinib have also been produced [23]. These inconsistent results may arise from variable use of radiation in these studies [24,25,26].

**Figure 1:**
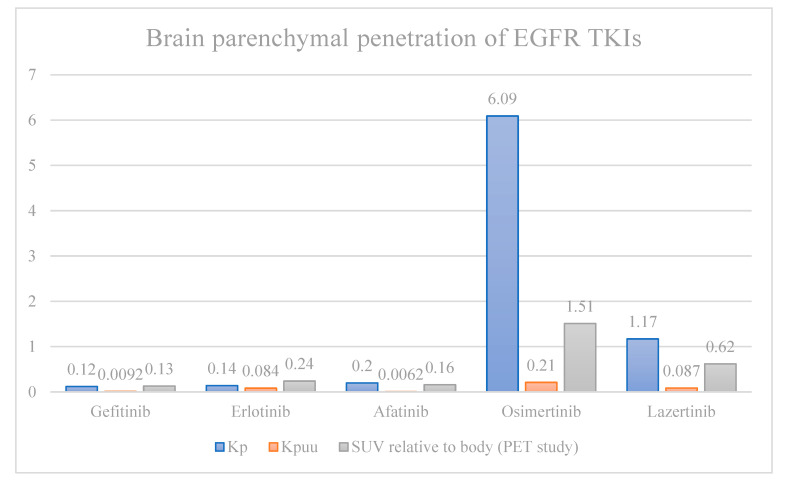
Brain parenchyma-to-plasma concentration ratio of total (Kp) and unbound (Kpuu) *EGFR*-directed TKIs in rats, and PET standardized uptake values relative to systemic uptake (SUV) in macaques. Kp and Kpuu are fundamentally different methods of measuring drug concentration compared to SUV, and should not be directly compared. Furthermore, direct comparison between Kp and Kpuu (rats) with SUV (macaques) is limited by interspecies limitations.

The efficacy of first- and second-generation *EGFR^mut^* TKIs is likely limited by sub-optimal CNS penetration when compared to third-generation drugs [24,25,26]. In a direct comparison of CNS penetration rates in rat models, afatinib, erlotinib, and gefitinib penetrated the brain parenchyma at only ~2–3% the rate of osimertinib and ~10–17% the rate of lazertinib, both of which are third-generation *EGFR^mut^* TKIs. All were dosed at 10 mg/kg. Even after adjusting for tissue binding, brain penetration was higher with third-generation TKI (Figure 1). A notable exception is rociletinib, a third-generation TKI with poor CNS penetration, which has not been granted FDA approval [27]. PET scans of radiolabeled TKIs showed osimertinib was the only one to reach significant brain exposures in macaques [25]. In animal studies, peak osimertinib brain-to-plasma concentration ratios are around 3.41, compared to 0.21 for gefitinib, and it reaches this level within 10–60 min, with persistence up to 21 days, compared to a peak of 6 h with undetectable levels at 24 h for gefitinib [26,27,28]. Afatinib, on the other hand, has such low CNS penetration that drug levels could not be detected in the brain [27]. Interestingly, osimertinib levels are highest in the gray matter, though existing data is inadequate to suggest a correlation between location of disease and treatment response [28].

#### 2.1.2. Third-Generation TKIs

**Figure 2:**
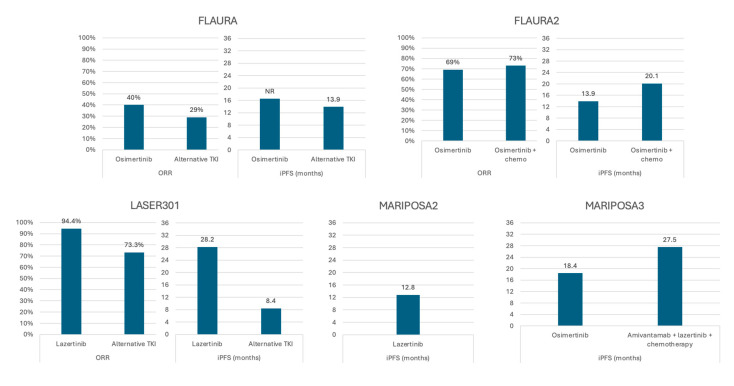
Response to third-generation *EGFR*-directed therapy in subsets of patients with central nervous system metastases—full analysis sets from multiple clinical trials. ORR—overall response rate; iPFS—intracranial progression-free survival.

Third-generation *EGFR* TKIs have significant advantages over earlier generations, in that they overcome the most common molecular mechanism of *EGFR* TKI resistance, the T790M mutation, and reach higher CNS concentrations, as detailed above [29]. Clinical and real-world data demonstrate that increased blood–brain barrier penetration translates to actual benefit (Figure 2).

Though not powered to specifically assess CNS response, the FLAURA trial supports the role of the third-generation TKI osimertinib in treating CNS disease. In this comparison of first-line osimertinib with earlier-generation TKIs in advanced *EGFR^mut^* NSCLC, subgroup analysis demonstrated a potential survival benefit with osimertinib in patients with baseline CNS metastases, with an effect size (HR 0.83) similar to the group without BMs (HR 0.79) and the overall group (HR 0.79). Though the confidence interval crossed equivalency, this subgroup included only 116 patients, and the magnitude of hazard reduction was promising [9]. Later analysis of the CNS subset showed improved overall response rate (ORR; 40% vs. 29%) and intracranial PFS (iPFS; NR vs. 13.9 months) (Figure 2) [30]. Similarly, trials of adjuvant osimertinib in resected, early-stage *EGFR^mut^* NSCLC showed 2- and 4-year BM-free survival rates of 98% and 92%, respectively, compared to 85% and 81% for the placebo group [31,32]. Most promising, pre-planned analysis of CNS disease response in patients with T790M mutations from the same trial demonstrated a response rate of 40% with osimertinib compared to 17% in a chemotherapy arm [33].

Retrospective data suggests BM response rates with osimertinib may be as high as 100%, with first response occurring as early as the first follow-up MRI or CT in 96% of patients, and best response by three months of treatment [34]. These responses appear to be durable as well, with median progression-free survival of 13.5 months and only 14% local recurrence at one year [35].

Despite evidence of improved efficacy of osimertinib in CNS metastases, mouse studies suggest that CNS-specific resistance to osimertinib can develop. Adua et al. found that cell lines developed from BMs resistant to osimertinib remain susceptible to the drug in vitro. After re-injecting these cell lines into mice, however, subsequent BMs quickly developed resistance. Systemic response was retained in these cell lines, which lack known resistance mutations, as was CNS penetration by osimertinib. They suggest that other collateral effects of metastatic progression confer some degree of drug resistance—for example, extracellular matrix changes—and repeat exposure to osimertinib further selects for these factors. Cell lines poised for osimertinib-resistance in the CNS tend to highly express *RHOC*, *SERPINE1*, *FOSL1*, and *PRNP*, which are upregulated by RhoA and Serum Responsive Factor (SRF) gene expression profiles [36]. In cases of LMD resistant to osimertinib, there is evidence of enrichment for *TP53*, *CDKN2A*, *RB1*, *NTRK1*, *MET*, and *CDK6* alterations in the CSF, but not the plasma [37]. It is unclear why differing resistance profiles develop in the two compartments, but it suggests progression in one compartment may not necessarily predict progression in the other. Therefore, continuing TKI therapy with addition of other active agents may be a viable treatment option at isolated CNS or systemic progression. Another consideration is changing to other later-generation *EGFR^mut^* targeted therapies that may avoid these resistance mechanisms.

To our knowledge, gene profiling of CNS metastases is not widely employed and there have been no experimental or clinical attempts at targeting these unique gene expression and alteration profiles seen in resistant CNS disease. With the emergence of *NTRK* and *MET* inhibitors and the ever-expanding menu of targeted therapies, future research should determine the importance of profiling progressive CNS disease. This will, however, be reasonably limited by the safety and technical limits of sampling brain metastases and the low yield of genetic material from CSF sampling.

There are several other third generation *EGFR^mut^* TKIs that appear to have similar efficacy in CNS disease, which are not approved by the United States Food and Drug Administration, but are available elsewhere. Furmonertinib, approved in China, demonstrated a 100% response rate in retrospective analysis of 26 patients with measurable BMs, and in clinical trials demonstrated better PFS compared to gefitinib in the subgroup with BMs [38,39]. Almonertinib was similarly tested at escalated doses in patients with BMs and demonstrated efficacy in a single-arm study [40]. Another third-generation *EGFR^mut^* TKI, befotertinib, was compared as first-line therapy to the first-generation icotinib in a Chinese phase III study of locally advanced or metastatic NSCLC patients. Forty-seven of the 182 receiving befotertinib had BMs at baseline. Intracranial response rates were 70% vs. 50% with icotinib. Notably, patients with “unstable” brain or meningeal metastases were excluded [41].

Specific mention is owed to zorifertinib (AZD3759), a third-generation *EGFR^mut^* TKI which was specifically designed to penetrate the blood–brain barrier, doing so with 100% efficacy [42]. The phase III EVEREST trial randomized *EGFR^mut^* patients with non-radiated, asymptomatic brain metastases to zorifertinib or gefitinib/erlotinib. Median PFS was improved overall, and subgroup analyses suggested improved efficacy regardless of number or size of brain metastases [42]. The National Medical Products Administration (China) approval specifically carries an indication for patients with BMs [43].

#### 2.1.3. Lazertinib and Amivantamab

Lazertinib, as mentioned above, is a third-generation TKI with more robust CNS penetrance than earlier generation TKIs and which outperformed gefitinib, both systemically and in the CNS in phase III study [26,44,45]. Though survival data were not mature at time of study publication, subset analysis of the CNS disease cohort found significant, sustained benefit to lazertinib. Median iPFS with lazertinib was 28.2 months versus 8.4 months with gefitinib, and median duration of response was not reached, despite a trend towards larger and more numerous BMs in the lazertinib group (Figure 2) [45]. This is comparable in magnitude to real-world retrospective data of single-agent osimertinib [34,35].

Lazertinib has also been studied in combination with amivantamab, a bi-specific antibody targeted against both *EGFR^mut^* and mesenchymal–epithelial transition factor [46]. Though the large size of amivantamab would suggest poor CNS penetration, BM response was boosted by the addition of amivantamab to chemotherapy alone in patients carrying *EGFR* exon 20 insertions in the PAPILLON study [47]. Phase 2 data of 42 patients with new BMs or LMD presented at the American Society of Clinical Oncology 2024 meeting found the combination of amivantamab and lazertinib led to BM and LMD response rates of 40% and 23%, though times on treatment were low (3.9 months for new BMs and 8.1 months for LMD) [48]. Additionally, the MARIPOSA trial, a phase III comparison of lazertinib with amivantamab against osimertinib in untreated *EGFR^mut^* NSCLC, demonstrated better disease responses in patients with a history of BMs who received the combination therapy, although enrolled patients were required to have asymptomatic stable or definitively treated BMs [46]. Serial brain MRIs were obtained as part of the trial, but a CNS-specific analysis has yet to be reported at time of this publication. Despite these promising results, the treatment gap for patients with CNS progression on third-generation TKIs persists.

#### 2.1.4. Fourth-Generation TKIs

It is reported that up to 20% of *EGFR^mut^* NSCLC patients will develop a C797S mutation which confers resistance to first-through-third generation *EGFR^mut^*-targeted TKIs by inhibiting their ability to block the ATP-binding pocket of EGFR [49]. Fourth-generation TKIs overcome this via allosteric inhibition of ATP binding to EGFR, thus reducing activity. Though no fourth-generation *EGFR^mut^* TKIs have progressed beyond phase I/II study at the time of this writing, many demonstrate activity against CNS metastases in pre-clinical models. Several recent publications review the progress on fourth-generation *EGFR^mut^* TKIs, and provide commentary on their potential clinical utility [49,50].

#### 2.1.5. Combining TKIs with Chemotherapy

One proposed method to improve CNS response rates is the combination of *EGFR^mut^* TKIs and chemotherapy. For example, the addition of chemotherapy to gefitnib improved intracranial response rates to 85% from 63% with monotherapy in an open-label study, though patients with symptomatic BMs and any LMD were excluded [19]. The combination of osimertinib and chemotherapy in the front-line setting demonstrated a PFS benefit over osimertinib alone in patients with CNS disease at baseline, in the FLAURA2 trial (24.9 versus 13.8 months, hazard ratio 0.47) [51]. Of the 222 patients with baseline CNS disease in that study, the rate of complete or partial CNS response was 86% with the addition of chemotherapy, compared to 72% with osimertinib alone; though this difference does not persist when evaluating measurable lesions only (88% versus 87%), chemotherapy still produced a higher complete response rate (48% versus 16%) and improved progression-free survival (not reached versus 17.3 months, hazard ratio 0.40) [52].

Clinically, the addition of chemotherapy to frontline TKIs is difficult due to toxicity, vastly increasing the incidence of grade 3+ adverse events compared to either medication class alone. Additionally, overall survival data is immature [51,53]. This may be a reasonable option in patients with BMs, however, for whom the risk of additional toxicity is weighed differently and more aggressive upfront therapy is important.

For patients on single-agent osimertinib with CNS or non-CNS progression, adding chemotherapy may boost responses while helping control further progression. In 37 patients with CNS disease who progressed on osimertinib, only 9 had further CNS progression after addition of chemotherapy, and only 2 of 7 patients without baseline CNS disease later developed it on the combination [54].

Though not a TKI, combining chemotherapy with the anti-*EGFR^mut^* amivantamab improved intracranial PFS to 12.5 months versus 8.3 months with chemotherapy alone after progression on osimertinib in the phase III MARIPOSA-2 trial, implying the importance of maintaining *EGFR^mut^* inhibition [53]. Addition of an alternative third-generation TKI to amivantamab and chemotherapy did not provide further benefit (intracranial PFS 12.8 months) [53]. This is in contrast to the IMPRESS trial, where the addition of chemotherapy to gefitinib after progression on gefitinib alone did not improve PFS and led to worse overall survival, though this may just illustrate the limitations of first-generation TKIs [55,56].

#### 2.1.6. Radiotherapy

Although the above discussion illustrates the efficacy of *EGFR^mut^* TKIs in controlling BMs, patients will almost invariably progress. The FLAURA trial, for example, reported an estimated probability of CNS-only progression in 5% of osimertinib- and 18% of first generation-treated patients, at 6 months. Those rates were a not insignificant 8% and 24%, respectively, at 12 months [30]. Furthermore, the presence of symptomatic brain metastases at diagnosis are not uncommon and may warrant early local therapy for rapid control [1,2].

Both whole-brain radiotherapy (WBRT) and stereotactic radiosurgery (SRS) are well-established for management of diffuse and limited BMs in NSCLC patients and are used as adjuncts with a variety of therapies [reviewed in Nardone et al. 2023] [57]. SRS, in particular, is likely more effective than TKI therapy, or at least early-generation TKIs, in managing CNS disease, as it directly outperforms erlotinib (with or without chemotherapy) and WBRT (with or without erlotinib), improving median overall survival to 64 months compared to 26 months and 35 months, respectively [18]. It stands to reason then, that there is value in combining radiotherapy (RT) with *EGFR^mut^* TKIs, in both the upfront setting for control of symptomatic CNS disease and for isolated CNS progression while on TKI therapy.

Three meta-analyses from 2015 to 2017 explored the efficacy of concomitant TKI and radiation therapy. Soon et al. found, in their review of 12 first-line BM treatment studies, that, despite similar response rates between TKIs alone, WBRT with TKIs, WBRT alone, and SRS alone, survival was improved when adding WBRT to TKIs or sequencing SRS with a TKI versus a TKI alone [58]. Two subsequent meta-analyses by Jiang et al., and Zheng et al., had significant overlap in the studies included in their analyses, but similarly found about half the rate of survival events with concomitant RT and TKI, compared to TKI alone [59,60]. Both studies replicated the results after adjustment for study heterogeneity and sensitivity analysis. Contrarily, in an unmatched, multi-institutional retrospective study of 94 patients, osimertinib or rociletinib (another third-generation TKI) monotherapy demonstrated similar intracranial outcomes to combined TKI and RT [61]. However, these results are confounded by multiple factors: 28% of patients received prior radiation before TKI therapy, the TKI monotherapy group had smaller size of BM and trended towards fewer BMs, and patients receiving RT were more symptomatic at baseline, implying worse disease. In clinical practice, early RT is often employed for symptomatic or threatening BMs, and these patients are likely less well clinically. Though a higher rate of adverse events is seen with concomitant therapy, these tend to be less severe and may be unlikely to limit clinical utility [59].

Importantly, a significant number of NSCLC patients in TKI trials undergo CNS radiation prior to enrollment, and thus the encouraging rates of CNS control with TKI monotherapy might be overstated or influenced by some synergistic effect of the two therapies [62]. For example, the addition of erlotinib within two months of completing WBRT improves PFS compared to WBRT alone [18]. This same pattern was observed with afatinib in the combined analysis of the LUX-Lung trials—though not compared directly, PFS was 13.8 months if patients received WBRT prior to afatinib, versus 6.9 months without [21]. This same effect was not seen in the chemotherapy cohort that received WBRT. In the AURA3 trial of osimertinib, overall CNS response rates were 74% if receiving brain radiation within the preceding 6 months, compared to 34% otherwise [33]. Sub-group analysis of high-dose osimertinib (160 mg) in patients with CNS disease similarly demonstrated increased PFS with prior RT in patients with BMs [63]. And, though a conflicting study of gefitinib did not demonstrate survival benefit with prior radiation, RT was reserved for symptomatic, and thus likely more advanced, patients in this cohort [16].

Together, these results may indicate some additive or boosted effect with proper sequencing or temporal administration of TKIs and radiotherapy. Additionally, RT subsequent to TKIs can provide meaningful control of BMs. In an analysis of 37 patients with previously untreated BM initially managed on osimertinib alone, 10 underwent salvage SRS for local recurrence with subsequent 1-year recurrence rates of 0% [35].

Focusing on a particular sequence of events may over-complicate matters—if symptomatic BMs develop, they should be treated quickly and effectively with RT. In this setting it may be reasonable to temporarily hold or delay TKIs to avoid additional side effects without compromising outcomes. When asymptomatic BMs are detected at diagnosis, it may be reasonable to attempt osimertinib monotherapy and, if response is inadequate, start RT.

### 2.2. Leptomeningeal Disease

Unfortunately, outcomes and treatment data for LMD is sparse when compared to BMs. This rare complication is more common in NSCLC with *EGFR^mut^*, occurring in just under 10% of patients, while carrying a life expectancy of 3 months or less [5,13,14,15]. Understanding this disease process is made more difficult by frequent exclusion of LMD from clinical trials [19,46]. However, modern treatment strategies, including intrathecal chemotherapy, *EGFR^mut^* TKIs, and radiation may all signal a path towards better outcomes [64].

#### 2.2.1. TKI Therapy

In a study of 25 patients with LMD, in which 14 received erlotinib and 11 received gefitinib, erlotinib was associated with a higher rate of CNS conversion (64.3% vs. 9.1%) [14]. However, all patients received concomitant intrathecal chemotherapy, and only 21.4% of erlotinib patients were on TKIs prior to LMD, compared to 54.5% of gefitinib patients. The aforementioned study of “pulsatile” erlotinib also included three patients with isolated LMD, of which two experienced radiographic response [20]. An 11 patient prospective study of afatinib demonstrated an LMD overall response rate of only 27.3%, with a dismal median overall survival of 3.8 months [25].

Similar to parenchymal BMs, osimertinib has also shown more promise in treating LMD. In a pooled analysis of five retrospective and six phase I/II studies, the overall leptomeningeal response rate with osimertinib was 42% (33% in phase I/II and 50% in retrospective studies) with 93% disease control rate. Aggregated one-year overall survival rate was 59% among five assessable studies [65]. The AURA3 cohort was notable for seven patients with LMD, all in the osimertinib arm. Four achieved complete or partial response, and zero progressed [33]. Even among a population heavily pre-treated for BMs and LMD, osimertinib improved median overall survival to 17.0 months compared to 5.5 months for those not receiving the drug, and confers a 52% lower likelihood of death compared to earlier-generation TKIs [14]. Further, a review of the 22 patients with LMD included in the AURA studies revealed six complete responses and six partial responses for an overall response rate of 55%; of the ten non-responders, eight had stable disease for at least 6 weeks [66]. Progression-free survival for LMD treated with osimertinib ranges from 3.7 to 17.3 months across studies [37,65,66].

More recently, the phase II BLOSSOM study examined standard-dose osimertinib in 73 patients with progression of LMD after treatment with first- or second-generation *EGFR^mut^* TKIs. Median intracranial PFS and overall survival were 12.5 and 15.6 months, respectively; in the 64 patients evaluable for LMD response, rate of stable disease or better was 81.3% [27]. Phase I and II trials of osimertinib 160 mg for LMD, twice the normal dose, produced similar results, though dose-efficacy analysis performed by Ahn et al., suggests the 80 mg of osimertinib received in the AURA studies should provide sufficient CNS exposure to control LMD (Table 1) [63,66,67]. Furmonertinib has also been prospectively evaluated at higher doses for LMD (240 mg, rather than the typical 160 mg). In the 48 patients with LMD evaluated, disease control rate was 92.1%. Importantly, median times on treatment and overall survival were greater than 8 months, in line with modern treatments, without excess toxicity [68].

**Table 1:** Studies of leptomeningeal disease treated with osimertinib at 80 mg and/or 160 mg daily.

Study	Study Type	Patients (n)	Osimertinib Dose	LMD Outcome
Reungwetwanna et al., 2018 (FLAURA) [30]	Small retrospective cohort	5	80 mg	ORR 80% DCR 100%
Ahn et al., 2020 (AURA) [66]	Retrospective	22	80 mg	ORR 55% DCR 91%
Lee et al., 2020 [14]	Retrospective	351 (110 osi-treated)	80 mg and 160 mg (mixed dataset)	With osi: mOS 17.0 months Without osi: mOS 5.5 months
Park et al., 2020 [63]	Phase II	40	160 mg	DCR 92.5% ORR 12.5% 67.5% retained response at 6 months
Yang et al., 2020 (BLOOM) [67]	Phase I	41	160 mg	ORR 62% DCR 95% DOR 15.2 months
Zheng et al., 2021 [37]	Retrospective	Cohort 1 (LMD treated with osi)—45	80 mg	iPFS 9.6 months
Park et al., 2024 (BLOSSOM) [69]	Phase II	64	80 mg	ORR 51.6% DCR 81.3% DOR 12.6 months

Interestingly, limited data suggests the presence of a T790M-resistance mutation does not affect CNS outcomes in osimertinib-treated patients, and outcomes may actually be improved in patients with concomitant T790M and LMD [63]. Furthermore, in one series, 18 of 23 T790M patients with follow-up CSF genotyping lost the mutation in the CSF on progression and had worse survival [14,37]. Contrast this to patients treated with first-generation *EGFR^mut^* TKIs in whom T790M mutations occur at lower-than-expected rates, implying inadequate treatment effect [2]. While the significance of these findings is unclear, it may suggest that where first-generation TKIs do not demonstrate adequate activity in the CSF to induce resistance, third-generation TKIs may clear out entire clones. It is not entirely clear why first- and second-generation *EGFR^mut^* TKIs would be less effective, however, based on CSF sampling studies.

Though CSF levels of osimertinib, measured via lumbar puncture, are similar to the levels reached in the brain, afatinib, erlotinib, and gefitinib have all demonstrated much higher levels of CSF penetration relative to both osimertinib and their own levels in the brain parenchyma [24,26]. It is suggested that the same efflux transporters that remove first- and second-generation TKIs from the blood–brain barrier may actually transport those TKIs into the CSF. In cases of LMD resistant to osimertinib, there is evidence of enrichment for *TP53*, *CDKN2A*, *RB1*, *NTRK1*, *MET*, and *CDK6* alterations in the CSF, but not the plasma [37]. Perhaps first- and second-generation TKIs seem ineffective for LMD despite high levels of CSF penetration, due to patterns of resistance. These drugs may inhibit the initial development or progression of LMD, but once the disease develops a new expression pattern that helps evade the drug, effectiveness is quickly lost.

As regards amivantamab and lazertinib, the combination led to LMD response rates of 23% in a group of 42 phase 2 participants with new BMs or LMD [48].

#### 2.2.2. Addition of Chemotherapy in LMD

Data specifically on combination *EGFR^mut^* TKI swith concomitant systemic chemotherapy is lacking. The FLAURA2 study contained 18 patients with LMD, 13 in the osimertinib plus chemotherapy group and 5 in the osimertinib alone cohort. Nine (69%) versus two (40%), respectively, achieved any response, and five (38%) in the combination therapy group achieved CR, compared to one (20%) in the monotherapy group. Importantly, times of treatment were about twice as long in the combination group for LMD patients (25.2 months versus 12.0 months) [52].

#### 2.2.3. Radiotherapy in LMD

The aforementioned PFS benefit of RT followed by TKI therapy in BM has not been demonstrated as clearly in LMD. Retrospective data suggests RT alone is associated with a poorer prognosis compared to *EGFR^mut^* inhibition, with a HR for overall survival events of 0.36 with osimertinib compared to RT [70]. Most prospective data is limited by sample size. The AURA LMD dataset only included three patients with prior WBRT. Two of those patients completed radiation within 3 months of starting TKI therapy, with one experiencing partial response and the other progression at 6 weeks. The third completed WBRT over two years before TKI therapy and achieved complete response [66]. Response rates were similar between those who had received prior radiotherapy (55%) and those who had not (57%) in the BLOOM study of high-dose osimertinib, though these results did not separate the 15 patients who received WBRT from the 5 who received more targeted RT [67]. Similar findings were seen in the BLOSSOM study of single-agent osimertinib 80 mg, with ORR not significantly changed between those receiving prior RT and those not. There was a signal suggesting prior WBRT led to worse survival, though this may be complicated by the consideration that those who receive earlier radiation are more likely to have symptomatic disease [69].

In an assessment of high-dose osimertinib, 26 LMD patients received prior RT, of whom 15 received WBRT. Though there was a signal for PFS improvement with prior RT (9.1 months versus 7.4 months without), this finding was not statistically significant. Timing from RT to high-dose TKIs is not specified [63]. It thus remains unclear how the timing of radiotherapy in relation to *EGFR^mut^* TKIs and how the dose of *EGR^mut^* TKIs are relevant, if at all, to the efficacy of this combination.

#### 2.2.4. Intrathecal Chemotherapy

Unique to LMD management is the use of intrathecal chemotherapy. A retrospective study of LMD management in 80 NSCLC patients included 22 patients with *EGFR^mut^*. Though *EGFR^mut^* TKI use prior to LMD was not specified, 10 subsequently received osimertinib, 3 erlotinib, and 1 afatinib for management of LMD. The rest of the cohort received intrathecal chemotherapy, systemic immunotherapy, and radiation, in combination with the other treatments or alone. Six-month overall survival with *EGFR^mut^* inhibition was 64% compared to 57% with intrathecal chemotherapy, and there was no difference between the two on multivariate analysis [70].

Though a full analysis of intrathecal chemotherapy is outside the scope of this review, recent phase I and II data from China suggests that intrathecal pemetrexed is more effective than traditional agents such as methotrexate and cytarabine, and can be safely combined with *EGFR^mut^* TKIs [71,72]. Efficacy of the combination is unclear, however, as one prospective study found no significant difference in response rate or survival between those who did and did not receive intrathecal chemotherapy prior to single-agent osimertinib [69]. Timing between the interventions was not examined, and the drugs were not given concomitantly.

One study included 18 *EGFR^mut^* patients who had progressed on *EGFR^mut^* TKIs prior to developing LMD, including three who received osimertinib. While receiving intrathecal pemetrexed, 14 continued on osimertinib, 2 received other TKIs, and 2 did not receive TKIs. Only three patients progressed, including one on osimertinib, one on alternative TKIs, and one not receiving TKIs [72]. In a phase II study including 132 patients, including 111 who had prior exposure to third-generation TKIs, patients were continued on current therapy with addition of intrathecal pemetrexed for LMD [71]. The group was “mostly treated” with concomitant third-generation *EGFR^mut^* TKIs, though the number was not reported. Disease response and stabilization occurred in 80.3% and 14.4% of patients, respectively, with prolonged OS of 15.0 months in responders versus 4.0 months with stable disease. Grade 3–4 adverse events were minimal [71]. Osimertinib dose was not specified in either study, though a single case report suggests 160 mg with concomitant intrathecal pemetrexed is tolerable [73].

Similar to RT, it remains unclear if timing of intrathecal chemotherapy and *EGFR^mut^* TKIs, as well as the dose of *EGFR^mut^* TKIs, are relevant to the efficacy of the combination.

### 2.3. Management of Isolated Systemic Progression

**Figure 3:**
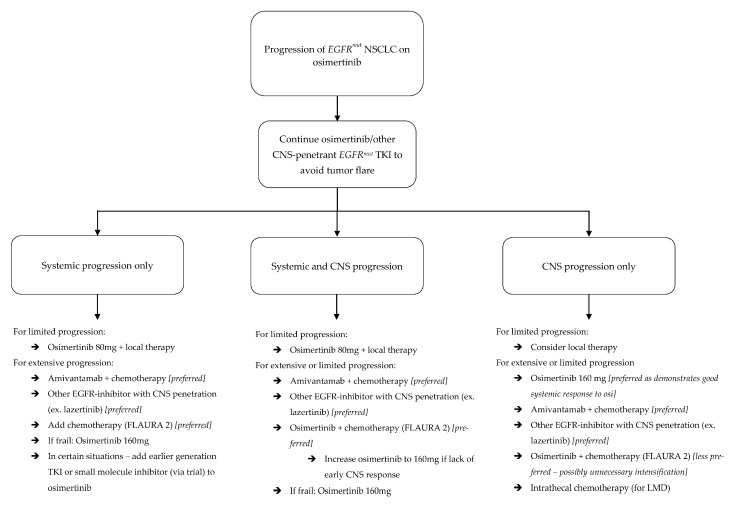
Algorithm for management of progression in *EGFR^mut^* NSCLC patients on osimertinib.

When disease progresses outside the CNS, alternative therapy is warranted (Figure 3); however, systemic TKI-resistance does not always infer CNS resistance. In the AURA LMD cohort, LMD PFS (11.1 months) was much longer, though not compared directly to, systemic PFS (4.4 months), implying continued sensitivity in one compartment, if not the other [66,67]. And, as discussed above, mechanisms of CNS resistance may differ from non-CNS resistance, or, at least, do not always develop in tandem; thus, if CNS disease continues to respond after non-CNS progression, there is an argument for continuing TKI therapy [36,37].

The question of combining modalities is especially important in situations where disease outside the CNS progresses but CNS disease is stable, as observational data suggests cessation of TKIs can lead to excessively rapid disease progression, known as disease flare. Chaft et al., evaluated a series of 61 patients in whom erlotinib or gefitinib were stopped after loss of clinical benefit; patients were enrolled in therapeutic trials and required a washout period of at least seven days at the end of TKI therapy. Fourteen (23%) of these patients experienced disease flare; of these, one of seven with baseline BMs had CNS progression and another three developed new BMs or LMD during the washout period [74]. In a review of 227 patients achieving at least three months of disease control on gefitinib or erlotinib, with a washout period of up to 21 days, disease flare occurred in 8.8% of patients. Of these 20 patients, 6 had CNS flare, all within 18 days or less [75].

**Figure 4:**
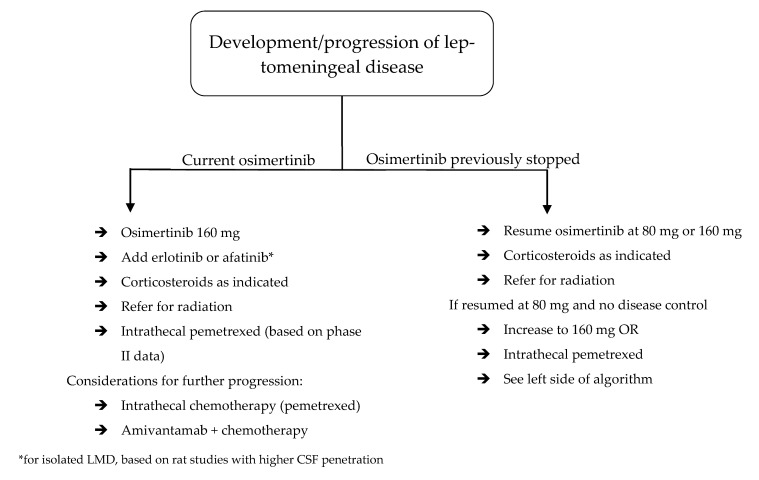
Algorithm for management of leptomeningeal disease in *EGFR^mut^* NSCLC patients.

More recently, a case of LMD flare after cessation of osimertinib, despite initiation of chemotherapy, demonstrated clinical response after resumption of osimertinib [76]. A separate case of LMD flare after switching osimertinib to chemotherapy was successfully treated with erlotinib, though this patient was not rechallenged with osimertinib [77]. In 49 patients with new or progressive LMD while on TKI therapy—the vast majority of whom received first- or second-generation drugs—TKI switch or dose intensification of the same drug led to clinical benefit in 60% overall and in all 4 who received osimertinib [78]. Though this is a different setting from that of stable CNS disease with progressive systemic disease, it illustrates the point that complete cessation of TKIs at progression may be premature. Furthermore, these observations, along with CSF penetration data of TKIs and experience with alternative treatment modalities, should guide management of isolated LMD progression (Figure 4).

Though to our knowledge there are no large published series of disease flare patterns after third-generation *EGFR^mut^* TKI withdrawal, it is reasonable to expect similar trends, or possibly higher rates of CNS flare, given better baseline CNS control with these drugs. Further, if local practice patterns dictate less aggressive radiotherapy in an era of widely-available CNS-penetrant TKIs, CNS flare post-TKI withdrawal may be more dramatic. Our group is currently evaluating CNS progression patterns after osimertinib cessation.

### 2.4. Management of Isolated Central Nervous System Progression

Progression in the CNS, however, is often morbid, and carries significant mortality risk. Management strategies can be derived from the data provided above (Figure 3).

In situations of limited progression, local therapy with RT, particularly SRS, is an important, and a recommended early consideration that leads to symptom control [79,80]. Both whole-brain radiotherapy (WBRT) and stereotactic radiosurgery (SRS) are well-established for management of diffuse and limited BM in NSCLC patients, and are used as adjuncts with a variety of therapies [57]. An,d as mentioned, SRS is likely more effective than *EGFR^mut^* in rapid control of BMs [18]. For more extensive disease, WBRT may be necessary if better disease control is desired at the risk of a higher likelihood of cognitive side effects, though this may be changing with more modern approaches to SRS [79,81,82].

Outside of radiation, multifocal CNS progression also warrants change in systemic therapy to an alternative with good CNS penetration. As detailed, molecular expression profiles in the CNS are different from those outside the CNS when osimertinib resistance develops [36,37]. This may also be the case when resistance develops to other drugs, and the treatment approach should seek to overcome resistance. If CNS progression develops during treatment with osimertinib or furmonertinib, one potential option is to increase the dosage [67,68]. This strategy may be particularly suited to frail patients who are already tolerating these options but may not be candidates for alternative intensified therapies. Other options include switching between CNS penetrant *EGFR^mut^* TKIs (ex: osimertinib to lazertinib) or switching from *EGFR^mut^* TKIs to an *EGFR*-targeted antibody (namely, amivantamab). Adding chemotherapy to TKIs may provide benefit with new BMs, but, as detailed above, this may not be effective with progressive LMD. All of the aforementioned strategies can be employed for progressive LMD, with the addition of intrathecal therapy (Figure 4).

## 3. Conclusions

As demonstrated, third-generation *EGFR^mut^*-targeted TKIs are effective in controlling CNS metastases in NSCLC, and should be used on the front line. What is less clear is the best approach for patients progressing within the CNS alone, or those only progressing outside the CNS, while on TKIs.

For CNS-only progression, radiation consistently provides progression and survival benefit and should be employed for those with symptomatic disease. It is unclear if TKI therapy still contributes to CNS disease control after a progression event, however. In the presence of multiple CNS metastases, progression of only one or two areas of disease suggest the TKI is controlling the rest, and withdrawal may lead to disease flare of those foci. Adua et al. demonstrated that CNS resistance to osimertinib is driven by upregulation of several genes that did not occur in non-progressive cell lines [36]. Thus, non-progressive CNS metastases may retain TKI sensitivity. However, the suggestion that extracellular matrix changes contribute to resistance implies resistance may be inducible in neighboring areas of disease. Once there is CNS progression on TKI therapy, progression of sub-clinical resistant disease may often be an unavoidable reality. More frequent imaging after CNS progression on TKI may be necessary and should be explored further.

Earlier- generation TKIs are strong substrates for common blood–brain barrier efflux transporters, and their relative lack of activity in brain metastases is likely a result of direct elimination [26]. These transport proteins have a much weaker affinity for osimertinib, however. As discussed above, prolonged exposure to osimertinib in the brain and CSF leads to the gradual development of drug resistance via changes in gene expression profiles [36,37]. Interestingly, the same transport proteins which eliminate TKIs from the brain space introduce them into the CSF; consequently, erlotinib and afatinib, which have very low brain penetration, have the highest CSF concentrations in human lumbar puncture samples [26]. Furthermore, gene expression profiles conferring resistance to osimertinib differ in the brain and CSF compartments, suggesting they may develop independently [36].

Altogether, multiple potential treatment-related conclusions are supported by these findings (Figure 3 and Figure 4). First, if osimertinib-treated patients maintain disease control in one compartment but not the other, such as the CSF or brain parenchyma, osimertinib may still be providing clinical benefit, and can be continued. Next, first- and second-generation TKIs may play a role in isolated LMD progression. Erlotinib in particular has pre-clinical and clinical data suggesting a role in LMD treatment, though this data is admittedly non-definitive [14,26]. If isolated LMD progression develops during osimertinib-based treatment, one strategy may be to add an *EGFR^mut^* inhibitor with good CSF penetration.

As regards systemic progression while CNS disease remains controlled, premature withdrawal of TKI for a less CNS-penetrant therapy, such as chemotherapy, may lead to tumor flare and have disastrous consequences. CNS disease flare after first-generation TKI withdrawal has been demonstrated, and we would expect similar, if not worse, outcomes after withdrawal of the more BBB-penetrant third-generation drugs. Osimertinib may also delay CNS disease development, and cessation without initiation of a similarly CNS-efficacious therapy may open the window for new CNS disease [31,32,74,75]. Future studies should consider subgroups of patients with CNS-only and non-CNS-only progression and their patterns of CNS progression after therapy change. Furthermore, upcoming clinical trials should consider at least maintaining TKI therapy until the start of the study regimen if CNS disease remains controlled.

While high dose osimertinib and amivantamab have demonstrated clinical efficacy, their high direct costs often exceed USD 300,000 and USD 700,000, respectively, per Quality-Adjusted Life Year gained [83,84]. This raises concerns about affordability and scalability, particularly in under-resourced settings. As far as routine CNS surveillance goes, serial MRI may not be feasible in many real-world contexts, due to imaging access and reimbursement challenges. Implementation strategies should incorporate cost-effectiveness analyses, payer coverage considerations, and equitable access frameworks, to ensure these advanced therapies are deliverable for the broader patient population.

## Abbreviations

The following abbreviations are used in this manuscript:

EGFR	Epidermal growth factor receptor
CNS	Central nervous system
LMD	Leptomeningeal disease
CSF	Cerebrospinal fluid
TKI	Tyrosine kinase inhibitor

## References

[1] Cagney D.N., Martin A.M., Catalano P.J., Redig A.J., Lin N.U., Lee E.Q., Wen P.Y., Dunn I.F., Bi W.L., Weiss S.E. (2017). Incidence and prognosis of patients with brain metastases at diagnosis of systemic malignancy: A population-based study. Neuro-Oncology.

[2] Kelly W.J., Shah N.J., Subramaniam D.S. (2018). Management of Brain Metastases in Epidermal Growth Factor Receptor Mutant Non-Small-Cell Lung Cancer. Front. Oncol..

[3] Fujita Y., Kinoshita M., Ozaki T., Takano K., Kunimasa K., Kimura M., Inoue T., Tamiya M., Nishino K., Kumagai T. (2020). The impact of EGFR mutation status and single brain metastasis on the survival of non-small-cell lung cancer patients with brain metastases. Neuro-Oncol. Adv..

[4] Ge M., Zhuang Y., Zhou X., Huang R., Liang X., Zhan Q. (2017). High probability and frequency of EGFR mutations in non-small cell lung cancer with brain metastases. J. Neurooncol..

[5] Iuchi T., Shingyoji M., Itakura M., Yokoi S., Moriya Y., Tamura H., Yoshida Y., Ashinuma H., Kawasaki K., Hasegawa Y. (2015). Frequency of brain metastases in non-small-cell lung cancer, and their association with epidermal growth factor receptor mutations. Int. J. Clin. Oncol..

[6] Shin D.Y., Na I.I., Kim C.H., Park S., Baek H., Yang S.H. (2014). EGFR mutation and brain metastasis in pulmonary adenocarcinomas. J. Thorac. Oncol..

[7] Bhatt V.R., D’sOuza S.P., Smith L.M., Cushman-Vokoun A.M., Noronha V., Verma V., Joshi A., Chougule A., Jambhekar N., Kessinger A. (2017). Epidermal Growth Factor Receptor Mutational Status and Brain Metastases in Non–Small-Cell Lung Cancer. JGO.

[8] Li W.-Y., Zhao T.-T., Xu H.-M., Wang Z.-N., Xu Y.-Y., Han Y., Song Y.-X., Wu J.-H., Xu H., Yin S.-C. (2019). The role of EGFR mutation as a prognostic factor in survival after diagnosis of brain metastasis in non-small cell lung cancer: A systematic review and meta-analysis. BMC Cancer.

[9] Ramalingam S.S., Vansteenkiste J., Planchard D., Cho B.C., Gray J.E., Ohe Y., Zhou C., Reungwetwattana T., Cheng Y., Chewaskulyong B. (2020). Overall Survival with Osimertinib in Untreated, EGFR-Mutated Advanced NSCLC. N. Engl. J. Med..

[10] Ju J.-S., Huang A.C.-C., Tung P.-H., Huang C.-H., Chiu T.-H., Wang C.-C., Ko H.-W., Chung F.-T., Hsu P.-C., Fang Y.-F. (2023). Brain metastasis, EGFR mutation subtype and generation of EGFR-TKI jointly influence the treatment outcome of patient with EGFR-mutant NSCLC. Sci. Rep..

[11] Huang A.C.-C., Huang C.-H., Ju J.-S., Chiu T.-H., Tung P.-H., Wang C.-C., Liu C.-Y., Chung F.-T., Fang Y.-F., Guo Y.-K. (2021). First- or second-generation epidermal growth factor receptor tyrosine kinase inhibitors in a large, real-world cohort of patients with non-small cell lung cancer. Ther. Adv. Med. Oncol..

[12] Liang S.K., Hsieh M.S., Lee M.R., Keng L.T., Ko J.C., Shih J.Y. (2017). Real-world experience of afatinib as a first-line therapy for advanced EGFR mutation-positive lung adenocarcinoma. Oncotarget.

[13] Li Y.-S., Jiang B.-Y., Yang J.-J., Tu H.-Y., Zhou Q., Guo W.-B., Yan H.-H., Wu Y.-L. (2016). Leptomeningeal Metastases in Patients with NSCLC with *EGFR* Mutations. J. Thorac. Oncol..

[14] Lee J., La Choi Y., Han J., Park S., Jung H.A., Su J.-M., Lee S.-H., Ahn J.S., Park K., Ahn M.-J. (2020). Osimertinib Improves Overall Survival in Patients With EGFR-Mutated NSCLC With Leptomeningeal Metastases Regardless of T790M Mutational Status. J. Thorac. Oncol..

[15] Cheng H., Perez-Soler R. (2018). Leptomeningeal metastases in non-small-cell lung cancer. Lancet Oncol..

[16] Yao Z.-H., Liao W.-Y., Ho C.-C., Chen K.-Y., Shih J.-Y., Chen J.-S., Lin Z.-Z., Lin C.-C., Yang J.C.-H., Yu C.-J. (2017). Real-World Data on Prognostic Factors for Overall Survival in EGFR Mutation-Positive Advanced Non-Small Cell Lung Cancer Patients Treated with First-Line Gefitinib. Oncologist.

[17] Goto K., Nishio M., Yamamoto N., Chikamori K., Hida T., Maemondo M., Katakami N., Kozuki T., Yoshioka H., Seto T. (2013). A prospective, phase II, open-label study (JO22903) of first-line erlotinib in Japanese patients with epidermal growth factor receptor (EGFR) mutation-positive advanced non-small-cell lung cancer (NSCLC). Lung Cancer.

[18] Gerber N.K., Yamada Y., Rimner A., Shi W., Riely G.J., Beal K., Yu H.A., Chan T.A., Zhang Z., Wu A.J. (2014). Erlotinib versus radiation therapy for brain metastases in patients with EGFR-mutant lung adenocarcinoma. Int. J. Radiat. Oncol. Biol. Phys..

[19] Hou X., Li M., Wu G., Feng W., Su J., Jiang H., Jiang G., Chen J., Zhang B., You Z. (2023). Gefitinib Plus Chemotherapy vs Gefitinib Alone in Untreated EGFR-Mutant Non–Small Cell Lung Cancer in Patients with Brain Metastases: The GAP BRAIN Open-Label, Randomized, Multicenter, Phase 3 Study. JAMA Netw. Open.

[20] Grommes C., Oxnard G.R., Kris M.G., Miller V.A., Pao W., Holodny A.I., Clarke J.L., Lassman A.B. (2011). “Pulsatile” high-dose weekly erlotinib for CNS metastases from EGFR mutant non-small cell lung cancer. Neuro-Oncology.

[21] Schuler M., Wu Y.-L., Hirsh V., O’bYrne K., Yamamoto N., Mok T., Popat S., Sequist L.V., Massey D., Zazulina V. (2016). First-Line Afatinib versus Chemotherapy in Patients with Non-Small Cell Lung Cancer and Common Epidermal Growth Factor Receptor Gene Mutations and Brain Metastases. J. Thorac. Oncol..

[22] Jung H.A., Woo S.Y., Lee S.-H., Ahn J.S., Ahn M.-J., Park K., Sun J.-M. (2020). The different central nervous system efficacy among gefitinib, erlotinib and afatinib in patients with epidermal growth factor receptor mutation-positive non-small cell lung cancer. Transl. Lung Cancer Res..

[23] Gijtenbeek R.G.P., Damhuis R.A.M., Groen H.J.M., van der Wekken A.J., van Geffen W.H. (2020). Nationwide Real-world Cohort Study of First-line Tyrosine Kinase Inhibitor Treatment in Epidermal Growth Factor Receptor-mutated Non-small-cell Lung Cancer. Clin. Lung Cancer.

[24] Togashi Y., Masago K., Masuda S., Mizuno T., Fukudo M., Ikemi Y., Sakamori Y., Nagai H., Kim Y.H., Katsura T. (2012). Cerebrospinal fluid concentration of gefitinib and erlotinib in patients with non-small cell lung cancer. Cancer Chemother. Pharmacol..

[25] Tamiya A., Tamiya M., Nishihara T., Shiroyama T., Nakao K., Tsuji T., Takeuchi N., Isa S.-I., Omachi N., Okamoto N. (2017). Cerebrospinal Fluid Penetration Rate and Efficacy of Afatinib in Patients with EGFR Mutation-positive Non-small Cell Lung Cancer with Leptomeningeal Carcinomatosis: A Multicenter Prospective Study. Anticancer. Res..

[26] Colclough N., Chen K., Johnström P., Strittmatter N., Yan Y., Wrigley G.L., Schou M., Goodwin R.J., Varnäs K., Adua S.J. (2021). Preclinical Comparison of the Blood–brain barrier Permeability of Osimertinib with Other EGFR TKIs. Clin. Cancer Res..

[27] Ballard P., Yates J.W.T., Yang Z., Kim D.-W., Yang J.C.-H., Cantarini M., Pickup K., Jordan A., Hickey M., Grist M. (2016). Preclinical Comparison of Osimertinib with Other EGFR-TKIs in EGFR-Mutant NSCLC Brain Metastases Models, and Early Evidence of Clinical Brain Metastases Activity. Clin. Cancer Res..

[28] Ekman S., Cselényi Z., Varrone A., Jucaite A., Martin H., Schou M., Johnström P., Laus G., Lewensohn R., Brown A.P. (2023). Brain exposure of osimertinib in patients with epidermal growth factor receptor mutation non-small cell lung cancer and brain metastases: A positron emission tomography and magnetic resonance imaging study. Clin. Transl. Sci..

[29] Zhao Z., Li L., Wang Z., Duan J., Bai H., Wang J. (2020). The Status of the EGFR T790M Mutation is associated with the Clinical Benefits of Osimertinib Treatment in Non-small Cell Lung Cancer Patients: A Meta-Analysis. J. Cancer.

[30] Reungwetwattana T., Nakagawa K., Cho B.C., Cobo M., Cho E.K., Bertolini A., Bohnet S., Zhou C., Lee K.H., Nogami N. (2018). CNS Response to Osimertinib Versus Standard Epidermal Growth Factor Receptor Tyrosine Kinase Inhibitors in Patients With Untreated EGFR-Mutated Advanced Non–Small-Cell Lung Cancer. JCO.

[31] Wu Y.-L., Tsuboi M., He J., John T., Grohe C., Majem M., Goldman J.W., Laktionov K., Kim S.-W., Kato T. (2020). Osimertinib in Resected EGFR-Mutated Non–Small-Cell Lung Cancer. N. Engl. J. Med..

[32] Herbst R.S., Wu Y.-L., John T., Grohe C., Majem M., Wang J., Kato T., Goldman J.W., Laktionov K., Kim S.-W. (2023). Adjuvant Osimertinib for Resected EGFR-Mutated Stage IB-IIIA Non–Small-Cell Lung Cancer: Updated Results from the Phase III Randomized ADAURA Trial. JCO.

[33] Wu Y.-L., Ahn M.-J., Garassino M.C., Han J.-Y., Katakami N., Kim H.R., Hodge R., Kaur P., Brown A.P., Ghiorghiu D. (2018). CNS Efficacy of Osimertinib in Patients with T790M-Positive Advanced Non-Small-Cell Lung Cancer: Data from a Randomized Phase III Trial (AURA3). J. Clin. Oncol..

[34] Imber B.S., Sehgal R., Saganty R., Reiner A.S., Ilica A.T., Miao E., Li B.T., Riely G.J., Yu H.A., Panageas K.S. (2023). Intracranial Outcomes of De Novo Brain Metastases Treated with Osimertinib Alone in Patients with Newly Diagnosed EGFR-Mutant NSCLC. JTO Clin. Res. Rep..

[35] Hui C., Qu V., Wang J.-Y., von Eyben R., Chang Y.-C., Chiang P.-L., Liang C.-H., Lu J.-T., Li G., Hayden-Gephart M. (2022). Local control of brain metastases with osimertinib alone in patients with EGFR-mutant non-small cell lung cancer. J. Neurooncol..

[36] Adua S.J., Arnal-Estapé A., Zhao M., Qi B., Liu Z.Z., Kravitz C., Hulme H., Strittmatter N., López-Giráldez F., Chande S. (2022). Brain metastatic outgrowth and osimertinib resistance are potentiated by RhoA in EGFR-mutant lung cancer. Nat. Commun..

[37] Zheng M.-M., Li Y.-S., Tu H.-Y., Jiang B.-Y., Yang J.-J., Zhou Q., Xu C.-R., Yang X.-R., Wu Y.-L. (2021). Genotyping of Cerebrospinal Fluid Associated With Osimertinib Response and Resistance for Leptomeningeal Metastases in EGFR-Mutated NSCLC. J. Thorac. Oncol..

[38] Yan N., Guo S., Huang S., Zhang H., Li X. (2024). The efficacy of furmonertinib in untreated advanced NSCLC patients with sensitive EGFR mutations in a real-world setting: A single institutional experience. Front. Oncol..

[39] Shi Y., Chen G., Wang X., Liu Y., Wu L., Hao Y., Liu C., Zhu S., Zhang X., Li Y. (2022). Furmonertinib (AST2818) versus gefitinib as first-line therapy for Chinese patients with locally advanced or metastatic EGFR mutation-positive non-small-cell lung cancer (FURLONG): A multicentre, double-blind, randomised phase 3 study. Lancet Respir. Med..

[40] Fan Y., Li H., Xu Y., Huang Z., Qin J., Zhou R., He J.-D., Zhu J., Yu S., Chen K. (2024). High-dose almonertinib in treatment-naïve EGFR-mutated NSCLC with CNS metastases: Efficacy and biomarker analysis. JCO.

[41] Lu S., Zhou J., Jian H., Wu L., Cheng Y., Fan Y., Fang J., Chen G., Zhang Z., Lv D. (2023). Befotertinib (D-0316) versus icotinib as first-line therapy for patients with EGFR-mutated locally advanced or metastatic non-small-cell lung cancer: A multicentre, open-label, randomised phase 3 study. Lancet Respir. Med..

[42] Zhou Q., Yu Y., Xing L., Cheng Y., Wang Y., Pan Y., Fan Y., Shi J., Zhang G., Cui J. (2025). First-line zorifertinib for *EGFR*-mutant non-small cell lung cancer with central nervous system metastases: The phase 3 EVEREST trial. Med.

[43] Alpha Biopharma Received NMPA Approval for Zorifertinib Tablets (Zorifer®), the World’s First EGFR-TKI for Lung Cancer with Brain Metastases. https://www.prnewswire.com/news-releases/alpha-biopharma-received-nmpa-approval-for-zorifertinib-tablets-zorifer-the-worlds-first-egfr-tki-for-lung-cancer-with-brain-metastases-302311486.html.

[44] Cho B.C., Ahn M.-J., Kang J.H., Soo R.A., Reungwetwattana T., Yang J.C.-H., Cicin I., Kim D.-W., Wu Y.-L., Lu S. (2023). Lazertinib Versus Gefitinib as First-Line Treatment in Patients With EGFR-Mutated Advanced Non–Small-Cell Lung Cancer: Results From LASER301. JCO.

[45] Soo R.A., Cho B.C., Kim J.-H., Ahn M.-J., Lee K.H., Zimina A., Orlov S., Bondarenko I., Lee Y.-G., Ni Lim Y. (2023). Central Nervous System Outcomes of Lazertinib Versus Gefitinib in EGFR-Mutated Advanced NSCLC: A LASER301 Subset Analysis. J. Thorac. Oncol..

[46] Cho B.C., Lu S., Felip E., Spira A.I., Girard N., Lee J.-S., Lee S.-H., Ostapenko Y., Danchaivijitr P., Liu B. (2024). Amivantamab plus Lazertinib in Previously Untreated EGFR-Mutated Advanced NSCLC. N. Engl. J. Med..

[47] Zhou C., Tang K.-J., Cho B.C., Liu B., Paz-Ares L., Cheng S., Kitazono S., Thiagarajan M., Goldman J.W., Sabari J.K. (2023). Amivantamab plus Chemotherapy in NSCLC with EGFR Exon 20 Insertions. N. Engl. J. Med..

[48] Yu H.A., Chen M.F., Hui A.B., Choudhury N.J., Lee J.J.-K., Zheng J., Ahn L.S.H., Pupo A., Nesselbush M., Jabara I. (2024). A phase 2 study of amivantamab plus lazertinib in patients with EGFR-mutant lung cancer and active central nervous system disease. JCO.

[49] Corvaja C., Passaro A., Attili I., Aliaga P.T., Spitaleri G., Del Signore E., de Marinis F. (2024). Advancements in fourth-generation EGFR TKIs in EGFR-mutant NSCLC: Bridging biological insights and therapeutic development. Cancer Treat. Rev..

[50] Zhang D., Zhao J., Yang Y., Dai Q., Zhang N., Mi Z., Hu Q., Liu X. (2025). Fourth-generation EGFR-TKI to overcome C797S mutation: Past, present, and future. J. Enzym. Inhib. Med. Chem..

[51] Planchard D., Jänne P.A., Cheng Y., Yang J.C.-H., Yanagitani N., Kim S.-W., Sugawara S., Yu Y., Fan Y., Geater S.L. (2023). Osimertinib with or without Chemotherapy in EGFR-Mutated Advanced NSCLC. N. Engl. J. Med..

[52] Jänne P.A., Planchard D., Kobayashi K., Cheng Y., Lee C.K., Valdiviezo N., Laktionov K., Yang T.-Y., Yu Y., Kato T. (2024). CNS Efficacy of Osimertinib With or Without Chemotherapy in Epidermal Growth Factor Receptor–Mutated Advanced Non–Small-Cell Lung Cancer. JCO.

[53] Passaro A., Wang J., Wang Y., Lee S.-H., Melosky B., Shih J.-Y., Azuma K., Juan-Vidal O., Cobo M., Felip E. (2024). Amivantamab plus chemotherapy with and without lazertinib in EGFR-mutant advanced NSCLC after disease progression on osimertinib: Primary results from the phase III MARIPOSA-2 study. Ann. Oncol..

[54] White M.N., Piotrowska Z., Stirling K., Liu S.V., Banwait M.K., Cunanan K., Sequist L.V., Wakelee H.A., Hausrath D., Neal J.W. (2021). Combining Osimertinib With Chemotherapy in EGFR-Mutant NSCLC at Progression. Clin. Lung Cancer.

[55] Soria J.-C., Wu Y.-L., Nakagawa K., Kim S.-W., Yang J.-J., Ahn M.-J., Wang J., Yang J.C.-H., Lu Y., Atagi S. (2015). Gefitinib plus chemotherapy versus placebo plus chemotherapy in EGFR-mutation-positive non-small-cell lung cancer after progression on first-line gefitinib (IMPRESS): A phase 3 randomised trial. Lancet Oncol..

[56] Mok T.S.K., Kim S.-W., Wu Y.-L., Nakagawa K., Yang J.-J., Ahn M.-J., Wang J., Yang J.C.-H., Lu Y., Atagi S. (2017). Gefitinib Plus Chemotherapy Versus Chemotherapy in Epidermal Growth Factor Receptor Mutation-Positive Non-Small-Cell Lung Cancer Resistant to First-Line Gefitinib (IMPRESS): Overall Survival and Biomarker Analyses. J. Clin. Oncol..

[57] Nardone V., Romeo C., D’iPpolito E., Pastina P., D’aPolito M., Pirtoli L., Caraglia M., Mutti L., Bianco G., Falzea A.C. (2023). The role of brain radiotherapy for EGFR- and ALK-positive non-small-cell lung cancer with brain metastases: A review. Radiol. Med..

[58] Soon Y.Y., Leong C.N., Koh W.Y., Tham I.W.K. (2015). EGFR tyrosine kinase inhibitors versus cranial radiation therapy for EGFR mutant non-small cell lung cancer with brain metastases: A systematic review and meta-analysis. Radiother. Oncol..

[59] Jiang T., Min W., Li Y., Yue Z., Wu C., Zhou C. (2016). Radiotherapy plus EGFR TKIs in non-small cell lung cancer patients with brain metastases: An update meta-analysis. Cancer Med..

[60] Zheng H., Liu Q.-X., Hou B., Zhou D., Li J.-M., Lu X., Wu Q.-P., Dai J.-G. (2017). Clinical outcomes of WBRT plus EGFR-TKIs versus WBRT or TKIs alone for the treatment of cerebral metastatic NSCLC patients: A meta-analysis. Oncotarget.

[61] Thomas N.J., Myall N.J., Sun F., Patil T., Mushtaq R., Yu C., Sinha S., Pollom E.L., Nagpal S., Camidge D.R. (2022). Brain Metastases in EGFR- and ALK-Positive NSCLC: Outcomes of Central Nervous System-Penetrant Tyrosine Kinase Inhibitors Alone Versus in Combination with Radiation. J. Thorac. Oncol..

[62] Fung A.S., Leighl N.B. (2019). Improving the Management of Brain Metastases in Oncogene-Addicted Non–Small-Cell Lung Cancer. JOP.

[63] Park S., Lee M.-H., Seong M., Kim S., Kang J.-H., Cho B., Lee K., Cho E., Sun J.-M., Lee S.-H. (2020). A phase II, multicenter, two cohort study of 160 mg osimertinib in EGFR T790M-positive non-small-cell lung cancer patients with brain metastases or leptomeningeal disease who progressed on prior EGFR TKI therapy. Ann. Oncol..

[64] Lee S.J., Lee J.-I., Nam D.-H., Ahn Y.C., Han J.H., Sun J.-M., Ahn J.S., Park K., Ahn M.-J. (2013). Leptomeningeal carcinomatosis in non-small-cell lung cancer patients: Impact on survival and correlated prognostic factors. J. Thorac. Oncol..

[65] Wen L., Zhen J., Shan C., Lai M., Hong W., Wang H., Ye M., Yang Y., Li S., Zhou Z. (2023). Efficacy and safety of osimertinib for leptomeningeal metastases from EGFR-mutant non-small cell lung cancer: A pooled analysis. Eur. J. Med. Res..

[66] Ahn M.-J., Chiu C.-H., Cheng Y., Han J.-Y., Goldberg S.B., Greystoke A., Crawford J., Zhao Y., Huang X., Johnson M. (2020). Osimertinib for Patients with Leptomeningeal Metastases Associated with EGFR T790M-Positive Advanced NSCLC: The AURA Leptomeningeal Metastases Analysis. J. Thorac. Oncol..

[67] Yang J.C., Kim S.-W., Kim D.-W., Lee J.-S., Cho B.C., Ahn J.-S., Lee D.H., Kim T.M., Goldman J.W., Natale R.B. (2020). Osimertinib in Patients with Epidermal Growth Factor Receptor Mutation-Positive Non-Small-Cell Lung Cancer and Leptomeningeal Metastases: The BLOOM Study. J. Clin. Oncol..

[68] Chen H., Yang S., Wang L., Wu Y., Wu Y., Ma S., He Z., Zhang C., Liu Y., Tang H. (2025). High-Dose Furmonertinib in Patients with EGFR-Mutated NSCLC and Leptomeningeal Metastases: A Prospective Real-World Study. J. Thorac. Oncol..

[69] Park S., Baldry R., Jung H.A., Sun J.-M., Lee S.-H., Ahn J.S., Kim Y.J., Lee Y., Kim D.-W., Kim S.-W. (2024). Phase II Efficacy and Safety of 80 mg Osimertinib in Patients with Leptomeningeal Metastases Associated with Epidermal Growth Factor Receptor Mutation–Positive Non–Small Cell Lung Cancer (BLOSSOM). JCO.

[70] Mills M.N., Uno A., Li P., Liveringhouse C., Kim Y., Oliver D.E., Perez B.A., Creelan B.C., Yu M., Forsyth P.A. (2024). Clinical Outcomes of Patients with Non-Small Cell Lung Cancer Leptomeningeal Disease Following Receipt of EGFR-Targeted Therapy, Immune-Checkpoint Blockade, Intrathecal Chemotherapy, or Radiation Therapy Alone. Clin. Lung Cancer.

[71] Fan C., Jiang Z., Teng C., Song X., Li L., Shen W., Jiang Q., Huang D., Lv Y., Du L. (2024). Efficacy and safety of intrathecal pemetrexed for TKI-failed leptomeningeal metastases from EGFR+ NSCLC: An expanded, single-arm, phase II clinical trial. ESMO Open..

[72] Li H., Zheng S., Lin Y., Yu T., Xie Y., Jiang C., Liu X., Qian X., Yin Z. (2023). Safety, Pharmacokinetic and Clinical Activity of Intrathecal Chemotherapy with Pemetrexed via the Ommaya Reservoir for Leptomeningeal Metastases from Lung Adenocarcinoma: A Prospective Phase I Study. Clin. Lung Cancer.

[73] Zhong W., Wu L., Huang L., Wang J., Shi H., Wu S. (2024). Double-dose osimertinib combined with intrathecal injection of pemetrexed improves the efficacy of EGFR-mutant non-small cell lung cancer and leptomeningeal metastasis: Case report and literature review. Front Oncol..

[74] Chaft J.E., Oxnard G.R., Sima C.S., Kris M.G., Miller V.A., Riely G.J. (2011). Disease flare after tyrosine kinase inhibitor discontinuation in patients with EGFR-mutant lung cancer and acquired resistance to erlotinib or gefitinib–implications for clinical trial design. Clin. Cancer Res..

[75] Chen H.-J., Yan H.-H., Yang J.-J., Chen Z.-H., Su J., Zhang X.-C., Wu Y.-L. (2013). Disease flare after EGFR tyrosine kinase inhibitor cessation predicts poor survival in patients with non-small cell lung cancer. Pathol. Oncol. Res..

[76] Takahashi T., Umeguchi H., Tateishi A., Yoshida T., Motoi N., Ohe Y. (2021). Disease flare of leptomeningeal metastases without radiological and cytological findings after the discontinuation of osimertinib. Lung Cancer.

[77] Kunimasa K., Mimura C., Kotani Y. (2017). Erlotinib Is Effective for Leptomeningeal Carcinomatosis due to Disease Flare after Osimertinib Treatment Failure. J. Thorac. Oncol..

[78] Flippot R., Biondani P., Auclin E., Xiao D., Hendriks L., Le Rhun E., Leduc C., Beau-Faller M., Gervais R., Remon J. (2019). Activity of EGFR Tyrosine Kinase Inhibitors in NSCLC With Refractory Leptomeningeal Metastases. J. Thorac. Oncol..

[79] Gaspar L.E., Prabhu R.S., Hdeib A., McCracken D.J., Lasker G.F., McDermott M.W., Kalkanis S.N., Olson J.J. (2019). Congress of Neurological Surgeons Systematic Review and Evidence-Based Guidelines on the Role of Whole Brain Radiation Therapy in Adults With Newly Diagnosed Metastatic Brain Tumors. Neurosurgery.

[80] Brown P.D., Jaeckle K., Ballman K.V., Farace E., Cerhan J.H., Anderson S.K., Carrero X.W., Barker F.G., Deming R., Burri S.H. (2016). Effect of Radiosurgery Alone vs Radiosurgery with Whole Brain Radiation Therapy on Cognitive Function in Patients with 1 to 3 Brain Metastases: A Randomized Clinical Trial. JAMA.

[81] Chang E.L., Wefel J.S., Hess K.R., Allen P.K., Lang F.F., Kornguth D.G., Arbuckle R.B., Swint J.M., Shiu A.S., Maor M.H. (2009). Neurocognition in patients with brain metastases treated with radiosurgery or radiosurgery plus whole-brain irradiation: A randomised controlled trial. Lancet Oncol..

[82] Yamamoto M., Serizawa T., Shuto T., Akabane A., Higuchi Y., Kawagishi J., Yamanaka K., Sato Y., Jokura H., Yomo S. (2014). Stereotactic radiosurgery for patients with multiple brain metastases (JLGK0901): A multi-institutional prospective observational study. Lancet Oncol..

[83] Lemmon C., Zabor E.C., Pennell N.A. (2021). Modeling the cost-effectiveness of adjuvant osimertinib in resected EGFR-mutant non-small cell lung cancer patients. JCO.

[84] Yue P., Zhang M., Feng Y., Gao Y., Sun C., Chen P. (2024). Cost-effectiveness analysis of amivantamab plus chemotherapy versus chemotherapy alone in NSCLC with EGFR Exon 20 insertions. Front. Oncol..

